# Prospective multicenter German study on pulmonary colonization with *Scedosporium* /*Lomentospora* species in cystic fibrosis: Epidemiology and new association factors

**DOI:** 10.1371/journal.pone.0171485

**Published:** 2017-02-08

**Authors:** Carsten Schwarz, Claudia Brandt, Elisabeth Antweiler, Alexander Krannich, Doris Staab, Sabina Schmitt-Grohé, Rainald Fischer, Dominik Hartl, Anja Thronicke, Kathrin Tintelnot

**Affiliations:** 1 Department of Pediatric Pneumology and Immunology, Cystic Fibrosis Center Berlin/Charité –Universitätsmedizin Berlin, Berlin, Germany; 2 Reference Laboratory for Cryptococcosis, Scedosporiosis and Imported Systemic Mycoses, FG 16, Robert Koch Institute, Berlin, Germany; 3 Biostatistics Unit, Berlin Institute of Health, Charité –Universitätsmedizin Berlin, Berlin, Germany; 4 Universitätsklinikum Bonn, Department of General Pediatrics, Bonn, Germany; 5 Privat Praxis West-München, Munich, Germany; 6 Universitätsklinikum für Kinder- und Jugendmedizin Tübingen, Department of General Pediatrics, Pediatric Hematology and Oncology, Tübingen, Germany; Lee Kong Chian School of Medicine, SINGAPORE

## Abstract

**Background:**

An increasing rate of respiratory colonization and infection in cystic fibrosis (CF) is caused by fungi of the *Scedosporium apiospermum* species complex or *Lomentospora prolificans* (*Sac-Lp*). These fungi rank second among the filamentous fungi colonizing the CF airways, after *Aspergillus fumigatus*. However, the epidemiology, clinical relevance and risk of pulmonary colonization with *Sac-Lp* are rarely understood in CF. The objective of the present prospective multicenter study was to study pathogen distribution and determine association factors of pulmonary *Sac-Lp* colonization in patients with CF.

**Material and methods:**

Clinical, microbiological and laboratory data of 161 patients aged 6–59 years with CF in Germany were analyzed for *Sac-Lp* distribution and association factors. The free statistical software R was utilized to investigate adjusted logistic regression models for association factors.

**Results:**

Of the 161 patients included in the study, 74 (56%) were male. The median age of the study cohort was 23 years (interquartile range 13–32 years). 58 patients of the total cohort (36%) were < 18 years old. Adjusted multivariate regression analysis revealed that *Sac-Lp* colonization was associated with younger age (OR 0.8684, 95%CI: 0.7955–0.9480, p<0.005) and less colonization with *H*. *influenzae* (OR 0.0118, 95%CI: 0.0009–0.1585, p<0.001). In addition, *Sac-Lp-*colonized patients had more often allergic bronchopulmonary aspergillosis (ABPA) (OR 14.6663, 95%CI: 2.1873–98.3403, p<0.01) and have been colonized more often with the mucoid phenotype of *Pseudomonas aeruginosa* (OR 9.8941, 95%CI: 1.0518–93.0705, p<0.05).

**Conclusion:**

Newly found association of ABPA and *Pseudomonas* revealed new probable risk factors for *Sac-Lp* colonization. Allergy might play a role in inducing immunologic host reactions which lead to a less effective response to species of *Sac-Lp*.

## Introduction

Cystic fibrosis (CF) is the most frequent genetic disease in the European Caucasian population. In Germany, approximately 8000 patients are affected with CF; each year almost 200 patients are newly diagnosed with CF. The disease is caused by mutations in the *CFTR* (*Cystic Fibrosis Transmembrane Conductance Regulator*) gene located on chromosome seven, which results in a defective chloride channel. This directly influences the airway surface liquid (ASL) the reduction of which leads to a defective mucociliary clearance and the production of sticky bronchial mucus. This in turn makes the patient susceptible to chronic airway inflammation and infections. This vicious circle causes lung tissue damage. While the clinical relevance of bacteria in airway infection such as *Pseudomonas aeruginosa* and *Staphylococcus aureus* has been well reviewed [1, 2], the role of filamentous fungi is poorly understood, except for *Aspergillus fumigatus*. During the last decade an increasing rate of bacterial and fungal colonization in the lungs of patients with CF and infection with some filamentous fungi belonging to the genus *Scedosporium* (family *Microascaceae*, division *Ascomycota*) was being recognized [3, 4]. Due to recent taxonomic changes, *Scedosporium apiospermum* is now considered to be a five-species complex, comprising *S*. *apiospermum sensu stricto*, *S*. *boydii* (formerly known as *Pseudallescheria boydii*), *S*. *aurantiacum*, *S*. *minutispora* and *S*. *dehoogii* [5, 6]. As recently examined by Lackner *et al*. [6], *Scedosporium prolificans* is genetically distinct from the other *Scedosporium* species, which explains that it was reassigned to the genus *Lomentospora* and is now called *L*. *prolificans*. Molds of the *Scedosporium apiospermum* complex and *L*. *prolificans*, referred to in the following text as *Sac-Lp* [7], are recognized as opportunistic pathogens and can cause infections such as sinusitis, pneumonia and disseminated infections primarily in patients with immunodeficiency [8–11]. With an overall estimated prevalence of up to 14%, *Scedosporium/Lomentospora* species rank second among the filamentous fungi colonizing the airways of patients with CF, after *A*. *fumigatus* [4, 7, 12]. In patients with CF, especially after long-term treatment, we see an emerging rate of *Scedosporium*/*Lomentospora* species colonizing the respiratory tract over months or even years [12–14]. As colonization with *Sac-Lp* may negatively influence the outcome of lung transplantation, such bronchopulmonary colonization is discussed as a contraindication for lung transplantation [9, 15]. *Sac-Lp* can be detected in various different environmental sources including water, soil and dung [3, 16]. As yet there is a lack of knowledge about the clinical relevance of and predisposing factors for respiratory colonization by *Scedosporium* species in patients with CF. The present study aimed at closing this gap of knowledge by prospectively evaluating epidemiology and associated factors in 161 patients, in 26 patients of which *Sac-Lp* has repeatedly been isolated from respiratory specimens. The primary objective of this study was to evaluate epidemiologic patterns and clinical relevance of respiratory colonization with *Sac-Lp* in patients with CF and to determine clinical association factors (possible predisposing factors) for respiratory colonization with these species in patients with CF. For this, samples from four distinct CF centers co-operating within the “German Network on *Scedosporium* and *Lomentospora* spp. in CF” founded in Germany in 2008 were examined. Currently (September 2016) 14 German centers for cystic fibrosis participate in this network.

## Material and methods

### Subject population and outcomes

The prospective cohort study was conducted by four German CF centers belonging to the “German Network on *Scedosporium* and *Lomentospora* spp. in CF”: Cystic Fibrosis Centers (1) Berlin, (2) Bonn, (3) Munich and (4) Tübingen. These CF centers cared for 726 patients during the study period, equivalent to 14% (13.9%) of all patients from the German CF Registry (in 2012: 5242). All individuals with confirmed diagnosis of CF were prospectively screened for *Sac-Lp* in the four CF centers from 09/2011 to 03/2015.

Patients who fulfilled the following criteria were included in the study:

1. complete data set of the following parameters
  - *CFTR* mutation
  - age
  - gender
  - FEV1/% predicted
  - pancreatic status
  - CF-related diabetes status
  - ABPA status
  - exacerbation p.a.
  - total IgE
2. minimum of 4 airway samples in 12 months
3. fulfilled criteria for pulmonary *Sac-Lp* colonization (i) detection of *Sac-Lp* in sputum, throat swab or bronchoalveolar lavage twice or more in 6 months, (ii) molecular-based confirmation of *Sac-Lp* in sputum, throat swab or BAL during study period in the reference laboratory.

Exclusion criteria were lung transplantation in the past, *Sac-Lp* isolation as a single (arbitrary) finding during the study period or missing written consent to enrollment in the German cystic fibrosis patient registry Muko.web.

Furthermore, patients with CF without repeated evidence of *Sac-Lp* colonization who were not lung transplanted were enrolled as control cohort. Clinical data of study population was retrieved from patients’ health records and the certified server-based patient registry Muko.web. All *Sac-Lp* isolates or respiratory samples from patients with suspected *Sac-Lp* colonization were sent to the Robert Koch Institute for precise species identification.

### Ethical issues

Written informed consent from patient or parent of patient has been obtained before enrollment in the study. Written consent was given as well to enrollment in the certified server-based German cystic fibrosis patient registry Muko.web (version 1.4.7). The study was approved by the local Charité –Universitätsmedizin Berlin Institutional Review Board (approval ID: EA2/080/11). We confirm that the work was done in accordance with the appropriate institutional review board and carried out by observing the ethical standards set forth in the Declaration of Helsinki; Recommendations guiding physicians in biomedical research involving human subjects. Adopted by the 18th World Medical Assembly, Helsinki, Finland, June 1964, amended by the 29th World Medical Assembly, Tokyo, Japan, October 1975, the 35th World Medical Assembly, Venice, Italy, October 1983, and the 41st World Medical Assembly, Hong Kong, September 1989.

### Primary endpoint

The primary outcome of the study was the determination of association factors of *Sac-Lp* colonization.

### Sample size determination

The design of the study included independent cases and controls with the above-mentioned inclusion criteria for statistical analysis. Furthermore, it was assumed that about two explanatory variables were required to yield good results for predicting the binary outcomes. According to Harrell et al. [17], a minimum of ten cases per variable were needed for logistic regression modelling to get a stable model. Thus, 20 events in the case cohort were required, leading to a total sample size of at least 50 patients for adjusted multivariate regression analysis.

### Statistical analysis

Multiple logistic regression analyses were performed to identify association factors of *Sac-Lp* colonization (yes, no). The following parameters have been adjusted in the multiple logistic regression analyses: age (in years), gender (male/female), predicted mean forced expiratory volume in one second (FEV1/predicted (%)), history of CF-related diabetes (CFRD) (yes, no), of allergic bronchopulmonary aspergillosis (ABPA) (yes, no), of exocrine pancreatic insufficiency (yes, no), number of annual exacerbations, deletion of phenylalanine at position 508 of the cystic fibrosis transmembrane conductance regulator gene (*CFTR*delF508: homozygous, non-homozygous), colonization with any bacterial and fungal species listed in Table 1 (each: yes, no). Patient with missing data were not included in the statistical analysis. Data distributions were checked by graphical inspections (box plots, QQ plots, histograms). Furthermore, distributions were arithmetically examined by skewness. All parameters were considered within the regression models and chosen for the final models by stepwise backward variable selection with AIC (Akaike information criterion). P values <0.05 were considered to be significant. All statistical analyses were performed using the free software R (Version 3.0.2, The R Project).

**Table 1:** Baseline characteristics of patients with CF without (control) and with (*Sac-Lp*) *Sac-Lp* colonization (total n = 161 patients).

Variable	Patients with CF	Control	*Sac-Lp*	P-value
Number of patients, n (%)	161 (100)	135(84)	26 (16)	
Age at enrollment, year, median (IQR)	23 (13, 32)	24 (14.00, 33.00)	20 (12.50, 27.50)	0.1927
Gender, male	74 (45.96)	58 (43)	16 (61.5)	0.09012
CFTR dF508 homozygous, n (%)	84 (52.17)	72 (53.3)	12 (46.2)	0.5275
FEV_1_/predicted (%), median (IQR)	77 (57.50, 99.00)	74.5 (57.00, 96.00)	96 (62.50, 114.00)	0.3516
Exocrine pancreatic insufficiency, n (%)	142 (88.19)	123 (91.1)	19 (73.1)	0.01677*
CF-related diabetes, n (%)	47 (29.19)	40 (29.6)	7 (26.9)	0.9798/1
ABPA, n (%)	35 (21.74)	24 (17.8)	11 (42.3)	0.009117**
Exacerbations p.a., median (IQR)	2.03 (3.00, 5.00)	2.04 (1.00, 3.00)	1.37 (1.00, 2.75)	0.1879
Total IgE, median (IQR) (kU/L)	65 (33.00, 100.00)	52 (18.50, 175.25)	92 (29.45, 202.50)	0.1703
*P*. *aeruginosa*, n (%)	116 (72.05)	93 (68.9)	23 (88.5)	0.05486
*P*. *aeruginosa* (mucoid), n (%)	74 (45.96)	59 (43.7)	15 (57.7)	0.2046
*S*. *aureus*, n (%)	133 (82.61)	110 (82.2)	22 (84.6)	1
*Burkholderia* spp., n (%)	6 (3.72)	5 (3.7)	1 (3.8)	1
*S*. *maltophilia*, n (%)	39 (24.22)	36 (26.7)	3 (11.5)	0.134
*A*. *xylosoxidans*, n (%)	24 (14.91)	22 (16.3)	2 (7.7)	0.3722
*H*. *influenzae*, n (%)	62 (38.51)	61 (45.2)	1 (3.8)	3.369e-05****
*A*. *fumigatus*, n (%)	64 (39.75)	59 (60.8)	5 (19.23)	0.02733*
Other *Aspergillus* spp., n (%)	11 (6.83)	11 (8.1)	0	0.2142
*C*. *albicans*, n (%)	105 (65.22)	93 (68.9)	12 (46.2)	0.04125*
Other *Candida* spp., n (%)	50 (31.06)	48 (35.6)	2 (7.7)	0.004688***

IQR: interquartile range, n: number, p.a.: per annum; %: percent; SD: standard deviation; OR: odds ratio

*p<0.05

**p<0.01

***p<0.005

****p<0.001.

### Data sources, assessment and definitions

Clinical data of the study population were retrieved from patients’ health records and Muko.web (updated version of Muko.doc). Colonization with bacteria or fungi was defined as at least one microorganism detected in respiratory secretions during the study period. But according to the definition of *Sac-Lp* colonization, two isolations in 6 months were needed. Annual pulmonary exacerbations according to Bilton et al. [18] were defined as average annual number of events within the study period. FEV1/predicted for *Sac-Lp-*colonized patients was defined as FEV1/predicted at first detection time point of *Sac-Lp* colonization. ABPA, CFRD and exocrine pancreatic insufficiency were defined as occurring between the date of diagnosis of CF and the date of the last routinely performed follow-up before statistical analysis. For the control cohort (Non *Sac-Lp* colonization), FEV1/predicted was defined as best FEV1/predicted during the study period. Clinically proven ABPA has been evaluated according to the diagnostic criteria of ABPA in patients with CF proposed by the Cystic Fibrosis Foundation Consensus Conference and the United Kingdom CF Trust.

### Analysis of pathogen colonization

Identification and susceptibility testing of all examined pathogens and *Sac-Lp* were routinely performed by the certified hospital-associated microbiological diagnostic unit. Growing of the cultures was performed according to quality standard guidelines [19]. Expectorated sputa and/or bronchoalveolar lavage from four participating centers were cultured on standard mycological media. Fungal colonies were initially microscopically pre-identified including examination of hyphal and spore characteristics. Precise species identification of *Sac-Lp* isolates was performed or confirmed by the Reference Laboratory for Scedosporiosis at the Robert Koch Institute, Berlin, Germany, by molecular methods such as sequencing of the ITS region of ribosomal DNA or species-specific hybridization on LCD microarray [20].

### Determination of ABPA

Proven ABPA has been evaluated according to diagnostic criteria of ABPA in patients with CF proposed by Cystic Fibrosis Foundation Consensus Conference and UK CF Trust [21, 22]. ImmunoCap assays for the analysis of total serum immunoglobulin E (IgE), specific *A*. *fumigatus* IgE and serological determination of *Af* antibodies were routinely performed by the certified hospital-associated microbiological diagnostic unit.

## Results

### Cohort characteristics

During the 563.5 person-years of follow-up, 726 subjects with CF visited the four CF centers. 26 patients with CF and confirmed *Sac-Lp* colonization were enrolled, and 135 patients with CF but without *Sac-Lp* at these four CF centers served as controls. For this study cohort (in total 161 patients) demographic, clinical and microbiological colonization characteristics are summarized in Table 1. 74 were male and 87 were female. The median age of patients was 23 years (interquartile range 13–32 years). The median FEV1/% predicted was 77 (range 57.50–99). The majority of subjects had exocrine pancreatic insufficiency (n = 142, 88.2%) and 29.2% had CFRD (n = 47). Delta F508 was the most frequent *CFTR* mutation with 88.81% (homozygous mutation 52.17%, heterozygous 36.65%, other genotypes 11.18). The median annual exacerbation rate of the total cohort was 2.03.

The percentage of co-colonized patients was different between patients harboring *Sac-Lp* and the control group. Table 1 gives an overview of bacterial and fungal isolates in the total, control and *Sac-Lp* cohort. In the total cohort Methicillin-susceptible *Staphylococcus aureus* (n = 133, 82.6%), non-mucoid *P*. *aeruginosa* (n = 116, 72.1%), mucoid *P*. *aeruginosa* (n = 74, 46%) and *Haemophilus influenzae* (n = 62, 38.5%) have been most frequently isolated, followed by *Stenotrophomonas maltophilia* (n = 39, 24.2%), *Achromobacter xylosoxidans* (n = 24, 14.9%) and *Burkholderia* species (n = 6, 3.7%). The most common fungi reported were *Candida albicans* (n = 105, 65.2%) and *A*. *fumigatus* (n = 64, 39.8%), followed by other *Candida* species (n = 50, 31.1%) and other *Aspergillus* species (n = 11, 6.8%).

### Prevalence of *Sac-Lp* colonization

In total, 29 patients (prevalence 4%) with CF from 4 German CF centers revealed confirmed *Sac-Lp* colonization during a time interval of 43 months (9/2011 to 3/2015), but three patients were excluded due to missing clinical data (exacerbation rate, total IgE).

### Characteristics of *Sac-Lp-*colonized patients

Table 1 shows demographic and clinical characteristics of patients that were colonized with *Sac-Lp* species. The *Sac-Lp* cohort (n = 26) consisted of 16 males (61.5%) and 10 females (38.5%) (Table 1). The median age of these patients was 20 years (interquartile range 12.5–27.5 years). In the *Sac-Lp* group delta F508 was the most frequent mutation with 76.92% (homozygous 46.15%, heterozygous 30.77% and others 23.07%).

In 19 (73.1%) patients a pancreatic insufficiency could be evaluated. CFRD was described in 7 (26.9%) patients. In 11 (42.3%) patients the diagnosis of an ABPA could be confirmed. The median number of annual exacerbations was 1.00 (interquartile range 1.00–2.75). Total IgE was elevated above 100 kU/L in 4/26 patients (15.4%) and in 5/26 patients (19.2%) above 500 kU/l. The median total IgE in patients with CF colonized with *Sac-Lp* was 92 kU/L (interquartile range 29.5–202.5).

The most frequently detected *Sac-Lp* species were *S*. *boydii* (17/26 patients, 65.4%) and *S*. *apiospermum* (15/26, 61.5%), while significantly less *L*. *prolificans* (2/26 patients, 7.7%) and *S*. *aurantiacum* (2/26 patients, 7.7%) were cultured (Table 2). Eight of 26 (30.8%) patients with *Sac-Lp* colonization showed pulmonary infection (data not shown). Overall, in 18 of 26 *Sac-Lp* patients only one *Sac-Lp* isolate was recovered from respiratory culture.

**Table 2:** Evidence of isolated Sac-Lp species per patient.

Variable	Number of patients (%)
Number of patients, n (%)	26 (100)
1 *species*	17 (65.4)
*S*. *boydii*	9 (34.6)
*S*. *apiospermum*	7 (26.9)
*S*. *aurantiacum*	1 (3.8)
2 *species*	8 (30.8)
S. boydii & *S*. *apiospermum*	6 (23.1)
*S*. *boydii* & *L*. *prolificans*	1 (3.8)
*S*. *apiospermum* & *S*. *aurantiacum*	1 (3.8)
3 *species*	1 (3.8)
*S*. *boydii* & *S*. *apiospermum* & *L*. *prolificans*	1 (3.8)

n: number, %: percent

Two different species within *Sac-Lp* were identified in 8/26 patients, while three species were found in only one of the 26 patients (Table 2).

### Association factors for *Sac-Lp* colonization

Unadjusted analysis revealed a highly significant negative correlation between *Sac-Lp* colonization and exocrine pancreatic insufficiency (p = 0.017), negative co-colonization with *A*. *fumigatus* (p = 0.027), with *A*. *xylosoxidans* (p = 0.046), with *H*. *influenzae*, *Hi* (p = 3.369e-05), with *C*. *albicans* (p = 0.041) and with other *Candida* species (without further specification) (p = 0.005) (Table 3). Another significant relationship was seen between *Sac-Lp* colonization and a positive correlation for ABPA (p = 0.009) (Table 3). No differences in the isolation of co-colonizing pathogens were found with respect to age, gender or other variables and *Sac-Lp* colonization.

**Table 3:** Adjusted odds ratios for association factors for Scedo colonization (total n = 26).

Variable	OR	LCL	HCL	P-value
Age at enrollment, year, median (IQR)	0.8684	0.7955	0.9480	0.0016***
Gender, male	2.2959	0.5808	9.0766	0.2360
CFTR dF508 homozygous, n (%)	0.8046	0.2151	3.0094	0.7467
FEV_1_/% predicted (median [IQR])	0.9932	0.9745	1.0123	0.4850
History of exocrine pancreatic insufficiency, n (%)	0.1571	0.0246	1.0040	0.0505
CF-related diabetes	0.8192	0.1547	4.3382	0.8146
History of CF-related diabetes, n (%)	0.3838	0.0371	3.9691	0.4217
History of ABPA, n (%)	14.6663	2.1873	98.3403	0.0057**
Exacerbations p.a. (median [IQR])	0.9471	0.6036	1.4862	0.8132
*P*. *aeruginosa*, n (%)	2.6586	0.2927	24.1467	0.3851
*P*. *aeruginosa* (mucoid), n (%)	9.8941	1.0518	93.0705	0.0451*
*S*. *aureus*, n (%)	1.2363	0.2099	7.2812	0.8146
*Burkholderia* spp., n (%)	3.1835	0.1014	99.9407	0.5102
*S*. *maltophilia*, n (%)	0.8124	0.1344	4.9089	0.8209
*A*. *xylosoxidans*, n (%)	0.3101	0.0372	2.5879	0.2794
*H*. *influenzae*, n (%)	0.0118	0.0009	0.1585	0.0008****
*C*. *albicans*, n (%)	0.2306	0.0498	1.0672	0.0605
Other *Candida* spp., n (%)	0.0151	0.0013	0.1812	0.0009****

Adjustment was performed with all indicated variables in the table, IQR: interquartile range, n: number, p.a.: per annum; %: percent; SD: standard deviation; vs: versus, OR: odds ratio, LCL: lower confidence limit, HCL: highest confidence limit

*p<0.05

**p<0.01

***p<0.005

****p<0.001.

Demographic, clinical and microbiological association factors correlating with *Sac-Lp* colonization calculated in an adjusted multivariate regression model are shown in Table 3. The adjustment of variables is specified according to criteria in the methods section. The following variables identified in univariate analysis were confirmed by adjusted multivariate analysis as being statistically significantly associated with *Sac-Lp* colonization: ABPA (OR 14.6663, 95%CI: 2.1873–98.3403, p = 0.0057), co-colonization with *Hi* (OR 0.0118, 95%CI: 0.0009–0.1585, p = 0.0008) and with *Candida* species (OR 0.0151, 95%CI: 0.0013–0.1812, p = 0.0009). Furthermore, adjusted multivariate analysis revealed that *Sac-Lp* colonization was associated with negative odds for age (OR 0.8684, 95%CI: 0.7955–0.9480, p = 0.0016). *Sac-Lp* patients were younger than Non-*Sac-Lp* patients (median of 18 years vs. median of 24 years). In addition, colonization with *Sac-Lp* was associated with increased odds for co-colonization with the mucoid phenotype of *Pa* (OR 9.8941, 95%CI: 1.0518–93.0705, p = 0.0451). A higher proportion of *Sac-Lp* patients (57.7% vs. 43.7%) revealed co-colonization with this pathogen compared to controls. However, the adjusted model did not confirm results from unadjusted univariate analysis for the correlation of exocrine pancreatic insufficiency, co-colonization with *A*. *fumigatus* and co-colonization with *C*. *albicans* with respect to *Sac-Lp* colonization. There were no significant differences in other baseline characteristics, including FEV1/predicted and annual exacerbations comparing *Sac-Lp* patients and Non-*Sac-Lp* patients.

## Discussion

Fungal complications such as lung deterioration and local inflammation in patients with CF are essentially caused by filamentous fungi. Colonization and infection with *Sac-Lp* in these patients is an emerging topic [8, 23]. The present multicenter study describes clinical association factors for *Sac-Lp* colonization in four German CF centers. Given the opportunity of inclusion of multiple German CF centers and selection on standardized highly selective SceSel+ agar [24], we calculated an overall prevalence of *Sac-Lp* of 4% which is in the range of previously reported German prevalences of 3.1% [7] and 5.3% [25], of French prevalence of 8.6% [26] and of Australian prevalences of 17.4% and 25% [3, 23].

In addition, the present multicenter work describes new association factors of pulmonary *Sac-Lp* colonization in CF. Adjusted multivariate analysis revealed that patients with *Sac-Lp* colonization showed significantly reduced co-colonization with *H*. *influenzae* and with *Candida* species and increased co-colonization with the mucoid phenotype of *P*. *aeruginosa*. Although the *in vitro* results by Kaur and colleagues showed that *P*. *aeruginosa* inhibits the growth of *S*. *aurantiacum* [27], our *in vivo* data could not confirm these findings. One reason could be the low prevalence of *S*. *aurantiacum* in Germany compared to other countries such as France [7, 28]. Furthermore, the adjusted model showed that patients harboring *Sac-Lp* were on average younger than non-colonized patients and had significantly more often ABPA. Correlation with acquisition of *Sac-Lp* at a young age may be explained by the fact that children tend to spend a lot of time outdoors. These outdoor activities may include a high risk of playing with *Sac-Lp-*contaminated water, soil and dung where these fungal pathogens can ubiquitously be found. A prospective 5-year-study of 128 French patients with CF aged 7.8 to 21 years confirmed acquisition of *Sac-Lp* at a young age, i.e. mean of 14.5 years although not recovered in children [26]. The median age of *Sac-Lp-*colonized patients of the present German work correlates well with the mean age of 73 *Sac-Lp-*colonized patients of another German study in 2011 (mean 22 years, range 6–51 years [7]), some of them overlapping with those of the current study.

Our results show an increased co-colonization with mucoid phenotype of *P*. *aeruginosa* in *Sac-Lp-*colonized patients compared to controls. This result is in contrast to earlier observations made by Blyth and colleagues [23] who found that mucoid *P*. *aeruginosa* colonization “protects” against *Sac-Lp* colonization in 12 *Sac-Lp*-colonized patients compared to 57 non-*Sac-Lp*-colonized patients with CF in Australia. However, the results of the Australian colleagues are based on unadjusted univariate analysis only which could not be confirmed by adjusted multivariate analysis. Furthermore, differences in results may possibly stem from limited size and older age (> 20 years) of the Australian *Sac-Lp* cohort. In addition, unlike Blyth and colleagues we did not just include a history of *P*. *aeruginosa* colonization but prospectively analyzed pathogen co-colonization during the study period.

We could show significantly decreased co-colonization with *H*. *influenzae* (*Hi*) in patients with *Sac-Lp* colonization. The “protective effect” of *Sac-Lp* colonization for *Hi* colonization was not dependent on age or gender since we adjusted for these among other factors in the present work. By looking for prediction or association factors of *Hi* colonization in CF, we found a significant positive correlation between exacerbation and *Hi* colonization (Table 4). *Hi* has the ability to form a biofilm structure in CF. Persistence of *Hi* strongly correlates with biofilm production [29]. Whether the reduced growth of *Hi* in *Sac-Lp* patients with CF is the result of additional antibiotic treatment (also against the increasingly colonized *Pa*) has to be examined in further prospective trials, including therapeutic assessment of *Sac-Lp-*colonized patients.

**Table 4:** Adjusted odds ratios for association factors of *H*. *influenzae* colonization (total n = 161 patients).

Variable	OR	LCL	HCL	P-value
Age at enrollment, year, median (IQR)	0.8563	0.7959	0.9212	0.0000
Gender, male	1.3453	0.4211	4.2978	0.6167
CFTR dF508 homozygous, n (%)	0.4049	0.1243	1.3192	0.1335
FEV_1_/% predicted (median [IQR])	0.9913	0.9742	1.0086	0.3202
History of exocrine pancreatic insufficiency, n (%)	9.5030	1.2902	69.9955	0.0271
CF-related diabetes, n (%)	0.6083	0.1641	2.2553	0.4572
History of ABPA, n (%)	0.2525	0.0563	1.1317	0.0721
Exacerbations p.a. (median [IQR])	1.5328	1.0917	2.1521	0.0136
*P*. *aeruginosa*, n (%)	0.6857	0.1471	3.1967	0.6310
*P*. *aeruginosa* (mucoid), n (%)	1.0013	0.1989	5.0407	0.9987
*S*. *aureus*, n (%)	13.5759	0.9586	192.2710	0.0538
*Burkholderia* spp., n (%)	0.8128	0.0364	18.1495	0.8959
*S*. *maltophilia*, n (%)	1.2035	0.3400	4.2598	0.7740
*A*. *xylosoxidans*, n (%)	0.1863	0.0318	1.0911	0.0624
*C*. *albicans*, n (%)	0.4447	0.1246	1.5871	0.2119
Other *Candida* spp., n (%)	0.3602	0.0798	1.6265	0.1843

IQR: interquartile range, n: number, p.a.: per annum; %: percent; SD: standard deviation; vs: versus, OR: odds ratio, LCL: lower confidence limit, HCL: highest confidence limit, adjustment was performed with all indicated variables in the table.

Colonization with *Hi* was associated with a decreased risk for filamentous *A*. *fumigatus* colonization [30]. Such a dichotomous colonization pattern for *Hi* and filamentous fungi (i.e. *Sac-Lp*) colonization has been observed as well in the present study. Besides the significance of *Sac-Lp* for post-transplant fungemia and infections [31], emerging significance of these organisms in relation to allergic bronchopulmonary mycosis has to be considered [26, 32–34]. In addition, Paugam and colleagues described a correlation of *Sac-Lp* colonization with ABPA [35]. These observations are now completed by confirming multivariate results of the present prospective multicenter study. We do know that *A*. *fumigatus* can sometimes cause APBA which is the most common fungal disease in this context and has well-reviewed clinical markers such as IgE, specific IgE for *A*. *fumigatus* and antibodies rAsp 4 and rAsp 6 for this disease [36, 37]. Also there is a well-established therapy for ABPA [22]. The pathomechanism for ABPA causing *Sac-Lp* colonization is not known, but we know *Aspergillus* itself is associated with a broad reaction of the innate and adaptive immune system that might influence the defense of *Sac-Lp* as described by Chortirmall and colleagues [38]. The known risk for the development of bronchiectasis after ABPA underlines the hypothesis of a damaged local host defense due to ABPA [39].

Despite our intriguing new findings, the cross-sectional design of the present work limits us in the causal interpretation of the relation between pulmonary *Sac-Lp* colonization and association factors found. In summary, analysis revealed that *Sac-Lp-*colonized patients had significantly more often ABPA and have been colonized more often with the mucoid phenotype of *P*. *aeruginosa*. In addition, *Sac-Lp* colonization was associated with younger age.

Based on our observations, we would suggest to regularly screen for ABPA in patients with *Sac-Lp* colonization. For further research it remains an interesting proposition to examine the chronological correlation of *Sac-Lp* colonization, the pathogen–pathogen interaction and the immunological host response to allergy and to infection or colonization with *Sac-Lp*.
